# In depth comparison of an individual’s DNA and its lymphoblastoid cell line using whole genome sequencing

**DOI:** 10.1186/1471-2164-13-477

**Published:** 2012-09-14

**Authors:** Dorothee Nickles, Lohith Madireddy, Shan Yang, Pouya Khankhanian, Steve Lincoln, Stephen L Hauser, Jorge R Oksenberg, Sergio E Baranzini

**Affiliations:** 1 Department of Neurology, University of California San Francisco, 513 Parnassus Ave, Room S-256, San Francisco, CA, 94143-0435, USA; 2 Complete Genomics, Inc, Mountain View, CA, USA

**Keywords:** Next generation sequencing, EBV transformation, Lymphoblastoid cell line, Genetics

## Abstract

**Background:**

A detailed analysis of whole genomes can be now achieved with next generation sequencing. Epstein Barr Virus (EBV) transformation is a widely used strategy in clinical research to obtain an unlimited source of a subject’s DNA. Although the mechanism of transformation and immortalization by EBV is relatively well known at the transcriptional and proteomic level, the genetic consequences of EBV transformation are less well understood. A detailed analysis of the genetic alterations introduced by EBV transformation is highly relevant, as it will inform on the usefulness and limitations of this approach.

**Results:**

We used whole genome sequencing to assess the genomic signature of a low-passage lymphoblastoid cell line (LCL). Specifically, we sequenced the full genome (40X) of an individual using DNA purified from fresh whole blood as well as DNA from his LCL. A total of 217.33 Gb of sequence were generated from the cell line and 238.95 Gb from the normal genomic DNA. We determined with high confidence that 99.2% of the genomes were identical, with no reproducible changes in structural variation (chromosomal rearrangements and copy number variations) or insertion/deletion polymorphisms (indels).

**Conclusions:**

Our results suggest that, at this level of resolution, the LCL is genetically indistinguishable from its genomic counterpart and therefore their use in clinical research is not likely to introduce a significant bias.

## Background

Epstein-Barr virus (EBV) is a herpesvirus that infects epithelial and B cells and has been associated with the development of various tumors, including Hodgkin’s and Burkitt’s lymphoma [1-4]. Since EBV is able to transform B cells into continuously proliferating lymphoblastoid cell lines (LCLs), it is commonly used as a tool in clinical research for creating an unlimited source of patients’ material [5-9]. Although LCLs have been used frequently as a source of DNA in genetic studies, controversy still exists about their reliability in faithfully replicating the variation present in the donor germ-line (e.g. [6,9-13]).

In all latently infected LCLs, the EBV genome is present in the form of extra-chromosomal copies (episomes) from which the viral genome is replicated [4]. Most research on the transforming abilities of EBV has been focused on the expression of viral gene products and the host’s transcriptional response. From this body of research it is now well understood that the viral transcription factor EBV nuclear antigen (EBNA)-2 activates the expression of several EBV proteins and non-coding RNAs - the growth transcription program - that interfere with the host’s signaling pathways [2,14]. Specifically, the growth transcription program drives cell transformation by activating cellular proliferation, while suppressing growth inhibitors [14]. Even though the viral gene products exert their transforming functions primarily by interacting with host proteins, recent evidence suggests that EBV also promotes genomic instability in the host [15]. Furthermore, EBV has the potential to cause mutations through integration and disintegration into the host’s genome (e.g. [16,17]). These putative early (pre-immortal) genetic consequences of EBV infection are less well studied. If ascertained, those structural genomic changes would have important implications for the interpretation of a large number of genetic studies that assume LCLs are a bona-fide source of genomic DNA.

In recent years, massively parallel DNA (i.e. next generation) sequencing has become increasingly affordable [18], enabling the discovery of causative DNA variants in rare genetic diseases [19-21] and providing new insights into carcinogenesis and autoimmunity [22-32]. A recent study reported the comparison of DNA isolated from peripheral lymphocytes and from an LCL from the same individuals by means of exome sequencing [33]. This study concluded that the exome fractions of genomic and lymphoblastoid cell line DNA (roughly 1-2% of the genomes) are more than 99% identical.

Whole genome sequencing can, in addition to identifying variation in coding regions, reveal copy number variation (CNV) and chromosomal rearrangements at high resolution. A whole genome sequence data set is therefore multilayered and can be queried for different aspects of genomic organization, making it a valuable tool for our study. Here we report the analysis of complete genome sequencing (40X) of a single individual to gain a better understanding of putative genetic alterations introduced by EBV transformation.

## Results

Here we report the complete sequencing and analysis of the normal genomic and lymphoblastoid cell line DNA from the same individual. We organized our analysis so as to proceed from investigation of major chromosomal rearrangements to small sequence variation, to single base changes. As a preliminary step, we performed high resolution karyotyping of the cell line to identify major chromosomal abnormalities. Fifteen out of 20 analyzed metaphases showed a normal karyotype (Additional file 1). Only 5 cells showed random changes in chromosome number, consistent with what would be expected for an early-passage LCL. Once sequencing data was obtained, we evaluated quality metrics of the two sequencing data sets, such as overall coverage, ratio of hetero-to-homozygous single nucleotide polymorphisms (SNPs) and the ratio of SNP transitions to transversions. These parameters are similar to those reported for previously sequenced genomes using the same technology (e.g. [20,21,34]) and other human datasets using next generation sequencing (Additional file 2). We also assessed the presence of viral DNA. To this end, we extracted raw reads of sequences that did not map to the human genome and aligned these to the EBV genome. We found an average of 2 copies of EBV DNA reads in the cell line as compared to 0 copies in the genomic DNA (data not shown), confirming that viral DNA was present only in the cell line sample. Since EBV has been described to integrate into host DNA (e.g. [35-40]), we also checked for viral DNA in all longer (>8 nt) insertions and substitutions that passed a pre-determined quality filter (SomaticScore of at least 0.1), as well as in all “non-reference” DNA stretches joining the two arms of a chromosomal rearrangement in the cell line (transition sequences). None of these calls supported the presence of inserted EBV genomic information.

**Figure 1:**
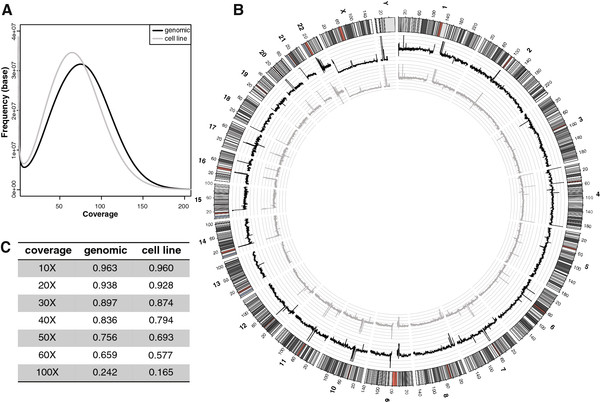
** Coverage of the two genomes. A**: Number of reads is plotted against the number of bases for which that number of reads was observed. The proportion of reads with a higher coverage (more reads) is slightly higher for the genomic DNA (black line) than for the cell line (grey line). **B**: Proportions of the two genomes at a certain X coverage. **C**: Regional plot of averaged normalized coverages of the genomic DNA (black line) and the cell line (grey line). The axes are spaced in increments of 10.

A total of 217.33 Gb of sequence were generated from the cell line and 238.95 Gb from the genomic DNA. This difference in coverage did not alter most of the parameters analyzed, as these were highly similar between the two genomes (Table 1). While the slightly deeper coverage of the genomic DNA did result in more variants being called in this sample, the overall depth of coverage was highly similar for the two genomes (~ 80% of both genomes were covered at least 40 times, Figure 1A and C). Also, the regional distribution of reads was highly concordant (Figure 1B). Once we established that quality measures were not significantly different, we set out to make a detailed comparison between the two genomes.

**Table 1:** Quality metrics of the sequenced genomes

	**genomic**	**cell line**
Gender	male	male
Gross mapping yield (Gb)	238.95	217.33
SNP Transitions/transversions	2.14076	2.1468
SNP het/hom ratio	1.582496107	1.571157051
INS het/hom ratio	1.344223031	1.289809921
DEL het/hom ratio	1.663654705	1.654600015
SUB het/hom ratio	1.715782613	1.713757678
SNP total count	3346813	3271797
INS total count	187392	170265
DEL total count	204807	186626
SUB total count	71344	66370
SNP novel rate	0.0487541	0.0472266
INS novel rate	0.189213	0.17794
DEL novel rate	0.239469	0.235096
SUB novel rate	0.309865	0.295857
Fully called genome fraction	0.967831529	0.961877785
Partially called genome fraction	0.004574129	0.006604011
No-called genome fraction	0.027594342	0.031518204
Synonymous SNP loci	9778	9387
Missense SNP loci	9329	8935
Nonsense SNP loci	90	89
Nonstop SNP loci	13	13
Frame-shifting INS loci	121	110
Frame-shifting DEL loci	108	100
Frame-shifting SUB loci	17	13
Frame-preserving INS loci	113	101
Frame-preserving DEL loci	107	91
Frame-preserving SUB loci	258	227
Frame-shifting/preserving ratio	0.514644351	0.53221957
Nonsyn/syn SNP ratio	0.954080589	0.951848301
Insertion/deletions ratio	0.914968727	0.912332687
Ins + del/SNP ratio	0.117185812	0.109081034
Coding insertion/deletions ratio	1.060465116	1.078534031
Coding SNP/all SNP ratio	0.00626387	0.006151054
Coding (ins + del)/all (ins + del) ratio	0.001129529	0.001112384

This table also lists information on the number and kind of indels identified per genome, but does not contain data on structural variances.

*Gb*: gigabase, *SNP*: single nucleotide polymorphism, *INS*: insertion, *DEL*: deletion, *SUB*: substitution, *(Non)syn*: (non-)synonymous.

**Figure 2:**
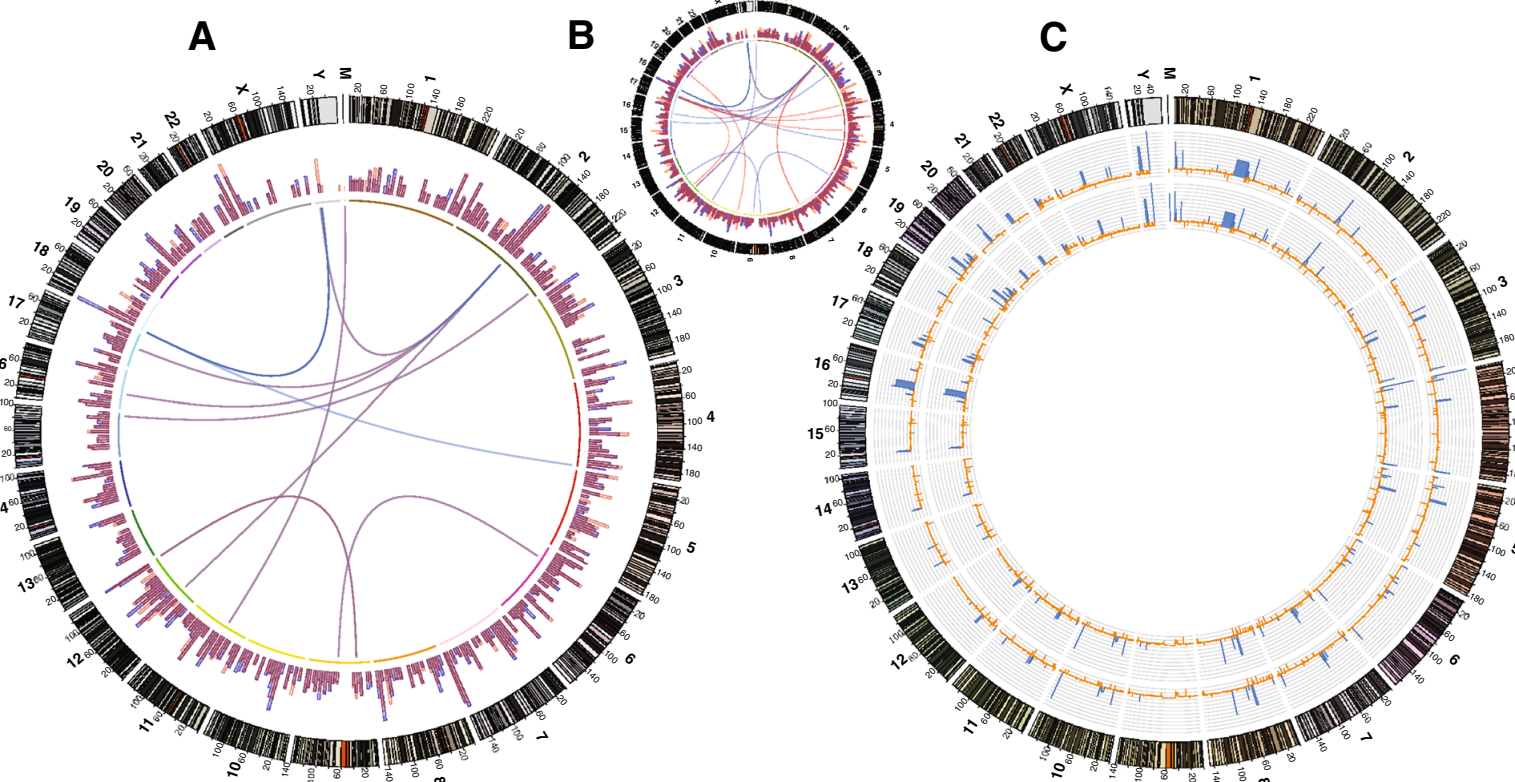
** Comparative display of structural variants of the two genomes. A**: For the outer circle, the number of chromosomal rearrangements was assessed in bins of 5 Mb for both the genomic (transparent blue) and cell line DNA (transparent red). In the inner circle inter-chromosomal rearrangements are displayed. Because of the transparent colors, overlaps between the two genomes appear in purple. **B**: For comparison, a similar visualization is shown as a smaller inset, plotting chromosomal rearrangements of the genomic DNA (blue) and the genome of an unrelated individual (red). **C**: Genome ploidies for the genomic and cell line DNA are shown in the outer and inner circle, respectively. The curve is colored in orange in genome stretches with a ploidy of smaller than 1, the area under the curve is filled blue when ploidy exceeds 2. The axes are spaced in increments of 0.5.

First, we assessed whether the two genomes differed in structural variants, i.e. chromosomal rearrangements and copy number variations (CNVs). Chromosomal rearrangements can be inferred from reads spanning over a stretch of DNA that is not contiguous in the reference genome (discordant reads). For example, if one half of a sequence read maps to chromosome 1 and the other half to chromosome 3, this might be indicative of a translocation event. By visual inspection of reported DNA junctions, we found that both genomes exhibited a largely similar number of chromosomal rearrangements compared to the reference and that they shared most of the inter-chromosomal junctions as well (Figure 2A). Indeed, a similar but small number of unique chromosomal rearrangements were identified in each genome (131 in genomic, 78 in cell line; see Additional files 3, 4. 5); 34% and 45% of these unique junctions, respectively, had been detected in more than 75% of all other genomes sequenced by CGI at that time and hence might represent false positives (Additional files 4 and 5). To examine whether observed junctions might be linked to the transformation process, we reasoned that if the observed dif-ferences were of biological origin (e.g. driven by the transformation), a set of genes involved in cell cycle regulation would be among the affected loci. GO and KEGG analyses revealed very similar gene categories affected by chromosomal rearrangements in both genomes (data not shown), thus suggesting these differences were likely random or false discoveries.

We next inspected ploidy (as a surrogate for CNVs) from genome coverage files in windows of 2 kb (Figure 2B). Both genomes shared almost all CNVs throughout the genome; of note, most DNA stretches with ploidy > 2 were observed near telomers and centromers, likely reflecting the difficulty to properly align reads in these highly repetitive DNA regions. The cell line showed a decreased copy number in only 4 regions (3 haploid regions, one deletion) as compared to the genomic DNA (Additional file 6). Four genes (KIAA0125, PRAME, ZNF280A, ZNF280B) and one pseudogene (ADAM6) were affected by the CNVs (Additional file 6). None of these is reported to have a negative impact on cell proliferation. Hence, it is unclear whether their reduced ploidy plays any role in the transformation process. Notably, a 9-fold increase in copy number of mitochondrial DNA was observed for the cell line, likely reflecting the increased energy demand of the actively dividing transformed cells (Figure 2B). This finding is consistent with a previous study [41].

**Figure 3:**
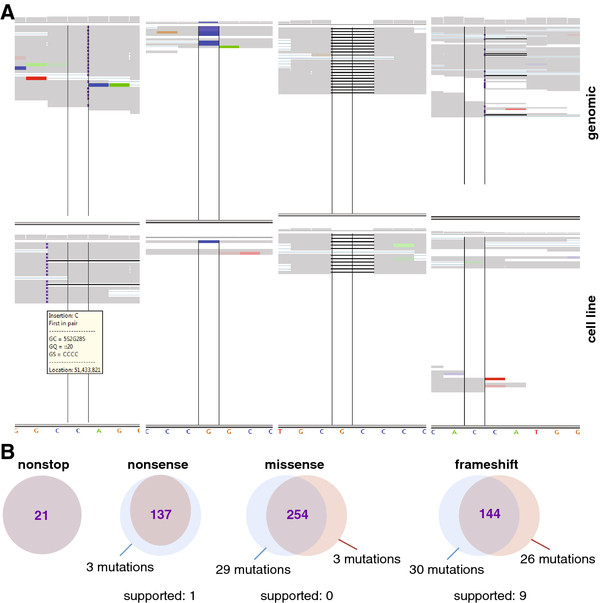
** Most variant calls within genes are shared between genomic DNA and cell line. A**: The Integrated Genomics Viewer (IGV) was used to assess variants in genes that were affected by non-synonymous mutations in both genomes, but where the number or the position of the variants differed. The following scenarios were encountered: (i) one of the calls in the two genomes was wrongly reported due to ambiguity in the alignments (left panel), (ii) coverage in one of the two genomes was very low at the called position, thus no variant call could be made (second left panel), (iii) a variant was determined homozygous in one of the genomes and heterozygous in the other, even though both genomes were homozygous (second right panel, “false positives”) or (iv) the two genomes looked really different (right panel, “true positive”). All reads were displayed in IGV. Each horizontal strip represents one read. Bases in agreement with the reference genome are displayed in grey, non-according bases are colored. Insertions are depicted by a purple square, deletions by a thick line, and gaps by a thin line. At the top of each panel, the relative coverage of each base in indicated by the height of the grey bar. The variant position is framed by two vertical lines. The genomic DNA is shown in the upper part of the panel, the cell line underneath it, at the bottom the reference sequence is displayed. **B**: For each class of non-synonymous variants (nonstop, nonsense, missense, frameshift), most mutations of this class in both genomic (blue) and cell line DNA (red) are shared between the two genomes (purple). A high percentage of the mutations called to be unique to either of the two genomes is not supported by actual reads.

We finally turned to an in-depth analysis of single nucleotide (SNP) and insertion/deletion (indel) polymorphisms. Using an automated whole-genome comparison algorithm (*calldiff* from cgatools) we found that 99.2% of the variant calls were identical between the two genomes (3,782,487 shared variants). Only 0.4% (15,364) and 0.3% (11,435) of variants were unique to the genomic and the cell line-derived DNA, respectively (Additional file 7, panel B). Of note, this level of discrepancy is within range of the error rate between technical replicates using CGI technology (SY, unpublished observations). Although the number of expected differences between the 2 genomes from the same individual was low *a-priori*, we continued searching for potentially functional differences, namely non-synonymous variants such as “missense” (amino acid changing mutation), “nonsense” (creating a stop codon where there was none before), “nonstop” (removing an existing stop codon) or “frameshift” (changing the reading frame of a gene). For each class of variant we identified the genes that were affected in each sample and then determined the overlap between the two genomes. While we found that 92% of the affected genes overlapped (5,995 genes total), these genes were not always affected by exactly the same mutations in both genomes. To test the reliability of these called differences, we inspected the sequence reads of 307 selected genes (exhibiting in total 647 non-synonymous variants) using the Integrative Genomics Viewer (IGV) [42]. We observed that in most instances local coverage in one of the two genomes was very low, thus no variation call could be made with high confidence at that position. In other cases, one of the calls in the two genomes was wrongly reported due to ambiguity in the alignments (i.e. the reads could have been aligned differently, so that the position of a variant differed between the genomes, even though the resulting non-reference sequence was the same). Another common discrepancy arose from the fact that a variant was determined to be homozygous in one of the genomes and heterozygous in the other, even though both genomes were homozygous (Figure 3A). Only 11% of the called differences between the two genomes were supported by visual inspection of actual reads (10 variants; Figure 3B), implying that a considerable fraction of the reported differences between genomic and cell line DNA represents false positives. 

**Figure 4:**
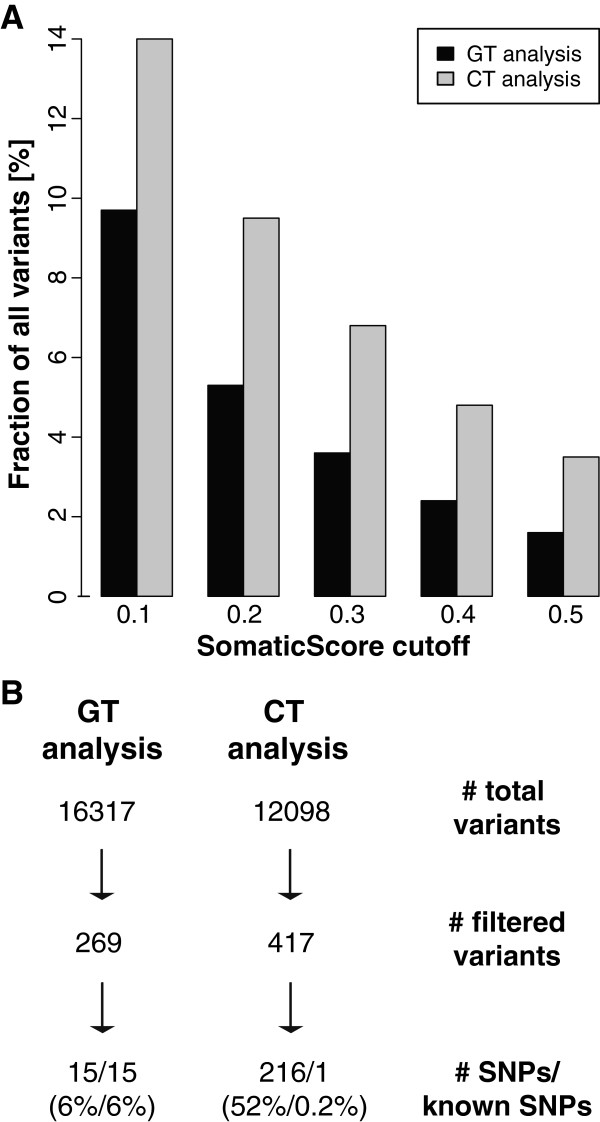
** Consequences of SomaticScore filtering in GT versus CT analysis. A**: Number of variants identified in GT (black bars) and CT (grey bars) analyses passing a certain SomaticScore filter, respectively. More variants meeting stringent filtering criteria are identified in the CT analysis. In addition, the ratio of the number of variants in CT to GT analysis is provided inside the graph. **B**: Some characteristics of the variants identified in GT and CT analyses, respectively. Even though a higher number of variants is found in the GT analysis, a lower number passes the SomaticScore filter of 0.5, as compared to the CT analysis. Among these are only a few SNPs (which are the most reliable called variants), all of which have records in dbSNP. In contrast, all variants identified in the CT analysis, represent SNPs and all of them, but one, have not been reported before.

In order to better control the false positive rate, we used the option -SomaticOutput within *calldiff*, in which a SomaticScore is computed for every variant that permits adjusting for sensitivity and specificity (sensitivity = 1 - SomaticScore). The SomaticOutput analysis requires specification of one sample as “normal” and another as “tumor” and generates an output containing all loci that are non-reference in the “tumor” sample. Since the transformed cell line can be regarded as a tumor sample derived from the normal genomic sample, definitions were set accordingly (“Cell line - > Tumor (CT)” analysis). The reciprocal definitions were also analyzed as a control (“Genomic - > Tumor (GT)” analysis). Since most of the variants only found in the genomic DNA sample are expected to be the result of sequencing or calling errors, the GT analysis provides a reasonable estimate of the experimental noise. For both comparisons, the number of variant calls was assessed using different SomaticScore cut-off values. As shown in Figure 4A, increasing the SomaticScore cut-off increased the proportion of CT to GT variants, thus potentially maximizing true positive findings. Even though the total number of variant calls unique to the genomic DNA (retrieved by the GT analysis) is larger (Figure 4B), a larger number of variants was detected in the CT analysis at all tested cut-off values (Figure 4A). To minimize false positives, we chose a stringent cut-off of 0.5 [at this level, the number of differences in the CT analysis (417) almost doubles those found in the GT analysis (269)]. Assessing the regional distribution of these mutations revealed that variants unique to the cell line were randomly distributed throughout the genome; in contrast, a high proportion of variants unique to the genomic DNA seemed within or near telomeric or centromeric regions (Additional file 8). Interestingly, 52% of variants in the CT analysis represented SNPs, compared to only 6% identified in the GT analysis (Figure 4B). Since SNPs are more reliably called than other classes of variants, they are less likely to constitute noise. This could explain the low fraction of (confidently called) SNPs in the GT analysis, which is expected to mainly represent technical noise. We next compared the proportion of SNPs that were novel (not present in the dbSNP database [43]) in both analyses. Strikingly, whereas all SNPs that were only present in the genomic DNA have been reported before, none of the SNPs unique to the cell line were annotated in dbSNP, with the exception of one variant (Figure 4B). Although the low number of identified variants between LCL and genomic DNA is within technical noise their novelty suggests that, if real, most of these differences would be random mutational events, driven by the accelerated proliferation of transformed cells. When assessing whether these SNPs altered coding sequences, we found that while 40% of them overlapped with genes, none had an impact on mRNA sequences. Specifically, except for one SNP, all variants are either located in introns or untranslated regions, thus their consequences are not straightforward (Additional file 9). None of the 15 SNPs unique to the genomic DNA fell within a gene. In order to estimate the exact error rate of this technology, we randomly selected 60 SNPs unique to the cell line and re-analyzed these by Sanger sequencing. We could not confirm any of the identified variants (data not shown), suggesting that the real number of differences between the two genomes is even smaller than that implied by the SomaticOutput analysis. 

Taken together, these results suggest that by using this technology at 40X resolution DNA from the cell line is mostly undistinguishable from genomic DNA from the same individual. The few putatively true differences are randomly distributed across the genome (Additional file 8) and do not seem to drive the transformation process.

## Discussion

Here we report on the first genome-wide sequence-based analysis of the immediate genetic consequences of EBV transformation on a low-passage lymphoblastoid cell line from a subject with MS. While genomes from MS and healthy individuals may differ slightly, we deemed that this would not affect the conclusions of our study, which focused on characterizing the genomic consequences of EBV transformation. These effects should be clear-cut and insensitive to the source of the sample (with the exception of certain tumors, where DNA may contain abundant somatic mutations). For decades, the cell-transforming abilities of EBV have been used in genetic research to create repositories of subjects’ DNA. While the roles of viral gene products in the transformation process have been described in detail, whether genetic alterations are introduced as a consequence of EBV transformation is less well understood.

Several studies have systematically compared LCLs directly to their parental cells using traditional molecular techniques such as SNP typing, gene expression, and whole chromosome analysis [12,44,45]. The overall consensus is that no reproducible differences were identified [7,45-47]. For instance, Redon et al. assessed differences between DNA from HapMap LCLs and their genomic counterparts and found that only 0.5% of observed CNVs were caused by transformation [46]. Another study confirmed that most CNVs in LCLs can also be seen in normal B cells [47]. Two of the CNVs reported in this latter study overlap with those detected in our LCL. This is not surprising since the genomic DNA was isolated from PBMCs, whereas the LCL is a B cell line – hence B cell specific CNVs were identified as differences to the genomic DNA in our study. The mitochondrial DNA CNV reported here was also seen as a “cell line-specific” CNV in a previous study [41].

Next generation sequencing was recently used to compare genomic and LCL DNA, although only the coding sequence (~1-2% of the genome) was assessed in that study [33]. Specifically, authors used exome sequencing to compare DNA from four LCLs with their corresponding genomic DNA (extracted from peripheral blood mononuclear cells). Focusing their analysis on SNPs and small indels, authors reported a 99.82% concordance between the parental DNA and the cell lines, with all discordant calls stemming from a single LCL-donor pair [33]. Given the relevance of non-coding regions in the modulation of gene expression and thus cell stability and function, whether the high level of concordance between genomic and LCL DNA extended to the whole genome remains an important question. By analyzing the whole genomes (at 40X) of an LCL and its genomic DNA counterpart, we identified 9,468 and 12,719 unique variants with respect to the reference genome, respectively. *In-silico* analysis reduced the number of differences to 417 (216 SNPs, 201 indels) in the LCL and 269 (15 SNP, 254 indels) in the genomic DNA. However, none of the 60 variants chosen for validation by Sanger sequencing were confirmed, thus suggesting that the number of real differences ought to be significantly smaller.

The known error rate of the ligation-based sequencing technique we employed was empirically determined to be approximately 1 in 100–200 kb (SY, unpublished observations). Hence, we can expect 20,000 to 30,000 errors in each genome. In a previous comparison of two technical replicate samples (sequencing the same sample twice) using the same technology, 27,893 differences were detected (SY, unpublished observations); we observed a similar number of differences (22,187) between the genomic DNA and the cell line genome in our analysis. These numbers highlight how close the difference between the two genomes is to technical noise. We therefore chose to minimize false positives in our analysis by applying stringent filters to the lists of called differences. By these means we identified a number of SNPs that seemed enriched in the cell line (216 SNPs in the CT analysis versus 15 SNPs in the GT analysis). These variants represented silent mutations and appeared to be random mutational events, possibly resulting from the accelerated division rate of the transformed cells. However, we were not able to confirm a selected subset of those SNPs and further validation is needed to establish any true discrepancies between the genomic DNA and the cell line.

## Conclusion

In conclusion, our results indicate that EBV transformed cell lines at low passage number/short time in culture are genetically indistinguishable to the parental cells, suggesting that discoveries in genetic studies conducted using low-passage LCLs with a normal karyotype can be extrapolated to the parental patient samples. We determined with high confidence that 99.2% of the genomes were identical, with no reproducible changes in structural variation (chromosomal rearrangements and CNV) or indels. While we identified 231 differences (216 from the LCL, 15 from the genomic DNA) in single nucleotide variants, none of the 60 randomly selected variants validated by Sanger sequencing. These findings suggest that the true differences between the two genomes ought to be less than 5.8% of the candidates identified by bioinformatics filters (proportions test; 95% confidence interval p-value: 1.3 x 10^-14^). We acknowledge that the sample size is a limiting factor of this study, and while similar results were reported by independent groups using different technologies [7,9,33,45-47], larger studies will be needed to precisely determine the genomic effects of EBV transformation.

## Methods

### Sample preparation

A lymphoblastoid cell line was established from whole blood from an adult male suffering from multiple sclerosis, essentially as described in [48] (a detailed protocol can be found in Additional file 10). Briefly, a buffy coat was obtained from freshly drawn blood and PBMCs were cultured with EBV supernatant. After infection, cells were kept in culture for 6 weeks. DNA was then extracted using standard salting-out procedure. DNA concentration was determined using the picogreen assay and adjusted to 0.1 μg/μl. DNA quality was assessed on an agarose gel prior to sequencing (Additional file 11). 15 μg DNA were sent for sequencing. The study was approved by the Institutional Review Board at the University of California San Francisco (UCSF).

### Sequencing

Genomes were sequenced and aligned by Complete Genomics Inc. (Mountain View, CA) (CGI, details on the technique in [34]). Sequence reads were aligned to genome release hg19 and all annotations were performed based on this genome release. CGI provided 6 data files (evidence, variants, gene variant summary, gene, CNV segments, and high-confidence junction files) that were used in the analysis (software version 1.10.0.22, format version 1.5; Additional file 7 panel A), most importantly the evidence and the variance files listing all sequenced loci that deviate from the reference genome. A position is either classified – or called – as “reference”, when the reads conform to the reference genome, or as “variant” if they do not accord. If a variant was called in this first round, corresponding reads are newly assembled to accurately determine the sequence of the variant locus; the resulting information is stored in the evidence file (assembled reads) and variance file (variant calls). Variants are classified into different classes, including SNP, deletion, insertion or substitution. All variants are assigned a score expressing the confidence of the variant call [this score was not used to prioritize variants for analysis; however, this score is incorporated into the SomaticScore (see below)]. Two other files, the gene variance summary file and the gene file, list all genes that are affected by a variant; the latter gives all variant positions that fall into genes, including untranslated regions, splice sites and introns, whereas the gene variance file specifies the number of mutations that can either be classified as “missense” (amino acid changing mutation), “nonsense” (creating a stop codon where there was none before), “nonstop” (removing a stop codon), “frameshift” (changing the reading frame of a gene) or “inframe”. Finally, the CNV segments file contains relative coverage and ploidy calls in windows of 2 kb, while the high-confidence junction file provides information on putative chromosomal rearrangement events. Genome data has been deposited at the European Genome-phenome Archive (EGA, http://www.ebi.ac.uk/ega/) which is hosted at the EBI under accession number EGAS00001000323.

### Genome analysis

#### Analysis of variant calls

The variant files of the two genomes were compared to each other using the function *calldiff* from cgatools 1.3.0 package [49]. An overview of overall differences was output by choosing the reports argument “SuperlocusStats”. For in-depth analysis of differences, the somatic output report was used (reports argument “SomaticOutput”). For this report, a “normal” and a “tumor” sample has to be specified. The tumor sample will be compared only to reference loci in the normal sample and the output will contain all non-reference loci in the tumor sample. Each call is assigned a “SomaticScore”, which indicates the reliability of the call. By using a SomaticScore of x, a sensitivity of 1-x is achieved. Being aware that the number of differences we observed was within the expected noise range, we chose a high SomaticScore of 0.5, knowingly losing some true positives, but minimizing false positives. From the resulting list of variants all loci that were not fully called were excluded. We did two analysis runs: (1) CT analysis (i.e. cell line is the tumor sample); (2) GT analysis, (i.e. genomic DNA is the tumor sample) as a comparison. Variants were mapped to genes using the CGI gene files.

#### Analysis of CNV calls

CNV calls were visually inspected by plotting the relative coverage of each genome using the *Circos* software [50]. For an in-depth analysis, the presence of calls reported in one genome, the “reference”, was assessed in the other genome, taking both cell line and genomic DNA as reference. For this search, only calls with a ploidyScore greater than 40 in the reference were considered. The start and end points of the CNV call are not required to be an exact match, but have to fall within a 2 kb window around the start and end points in the reference. For each CNV call present in both genomes, ploidy calls were compared. When these differed between the two genomes, the locus was output for both genomes and visually assessed.

#### Analysis of chromosomal rearrangements

CGI provides a high confidence junction file for each sequenced genome, which lists events of discordant read pairs within a given DNA nanoball (DNB). In this file, sequences that are not adjacent in the reference genome are reported; these are defined as “junctions”, consisting of a “left arm” reference sequence, a breakpoint with an optional transition sequence (a stretch of sequence that is not contained in the reference genome) and a “right arm” reference sequence at a non-adjacent genome location. CGI also reports the frequency with which each identified junction was found in previously sequenced genomes (the higher the frequency, the more likely the reported junction might be an artifact of the sequencing technology). For the graphical (e.g. *Circos*) analysis, all high confidence junctions seen in 75% or more of CGI sequence data sets were removed. For visually contrasting structural variance calls of the two genomes, only inter-chromosomal high confidence junctions (as determined by CGI) were plotted. In addition, the total number of junctions in bins of 5 Mb was calculated with the help of the software tool *binlinks* that is distributed together with *Circos*. In-depth analysis was performed by comparing CGI’s high confidence junctions files for the different genomes using cgatools 1.3.0 *junctiondiff*[49]. The program was run using the standard settings, i.e. the scoreThresholdA was set at 10, scoreThresholdB at 0, the maximum distance between the coordinates of the putatively compatible junctions at 200 and the minimum deletion length at 500. CGI provided accession numbers of genes that were affected by chromosomal rearrangements. Accession numbers affected by junctions specific to one of the genomes were translated into geneIDs and symbols using the Bioconductor/R package “biomaRt” [51]. Then, enrichment for GO and KEGG categories was assessed using the “GOstats” R package [52].

#### Analysis of non-synonymous variants

For each class of non-synonymous variant - “missense”, “nonsense”, “nonstop” or “frameshift” -, all genes with at least one mutation in genomic and cell line DNA were determined, respectively. Of all genes that were affected by the same class of mutation in both genomes, mutations were compared between genomic and cell line DNA. If position or type of the variant was not identical in the two genomes, raw sequence reads were displayed using the Integrative Genomics Viewer [42] and visually compared.

#### Detection of viral DNA

To assess the presence of viral DNA sequences within the whole genome data sets, all reads that could not be mapped to the human genome were extracted. Next, the reference genome sequence of EBV was downloaded in fasta format from the NCBI. Then, unmapped reads were aligned to this reference genome using *bwa*[53]. Subsequently, *samtools*[54] was used to convert aligned reads into bam format to enable display in IGV and calculate EBV genome coverage (44X). In addition, we extracted all transition sequences (sequences that join the two arms of chromosomal junctions, but are not present in the reference genome) as well as reported insertions and longer than 8 nucleotides from the junctions that were unique to the cell line and blasted them against the EBV genome (using NCBI BLAST).

### Karyotyping and Sanger sequencing

Karyotyping was performed by the Cytogenetics Laboratory of UCSF. Sanger Sequencing was performed by the Genomics Core Facility of UCSF. Sequencing primers were designed based on a 200 bp region flanking the SNP that was inquired. 60 randomly selected discordant SNPs were sequenced using two independent primer pairs per position.

## Competing interests

SY and SL are employees of Complete Genomics, Inc.

## Authors’ contributions

DN performed the analysis and drafted the manuscript. LM, SY, PK, SL and SEB contributed to the statistical analysis. SLH, JRO and SEB designed the study. SEB helped to draft the manuscript. All authors read and approved the final manuscript.

## Supplementary Material

Additional file 1 ** High resolution karyotype of cell line.** The cell line exhibits a normal karyotype. Click here for file

Additional file 2 ** Comparison with previously published whole exome/genome datasets.** Comparison of a number of sequencing characteristics with previously published genomes/exomes. Click here for file

Additional file 3 ** Number of chromosomal rearrangements in both genomes.** Information on all high confidence junctions identified per genome and the number of unique junctions, specifying the number of junctions with a frequency of less than or equal to 75% (i.e. seen in less than or exactly 75% of all genomes sequenced by CGI at the time) and the percentage of inter-chromosomal rearrangements. Click here for file

Additional file 4 ** Chromosomal rearrangements that were only reported for the genomic DNA sample.** This file contains details on the chromosomal rearrangements (junctions) unique to the genomic DNA sample. Click here for file

Additional file 5 ** Chromosomal rearrangements that were only reported for the cell line sample.** This file contains details on the chromosomal rearrangements (junctions) unique to the cell line sample. Click here for file

Additional file 6 ** Copy number variation (CNV) differences between genomic and cell line DNA.** Copy number variation (CNV) differences between genomic and cell line DNA. Click here for file

Additional file 7 ** Analysis strategy and overall differences found between the two sequenced genomes.A**. Workflow of the genome analysis. Besides various data files and software tools provided by Complete Genomics (blue), Bioconductor packages and R (green) were used for analysis. **B**. Gross comparison statistics as output from cgatools *calldiff* SuperlocusStats. “Identical” sequences are sequences that are fully called and identical in both genomes. “Consistent” sequences are not fully called, but what is called is identical in both genomes. “Only C” and “Only G” denote variants only found in cell line and genomic DNA, respectively. At “mismatch” positions, the two genomes are different from each other and different from the reference. “Phase-mismatch” means that even though the two genomes have the same alleles, the phase of the alleles differ. The two genomes don’t have any “ploidy mismatches” because genomes are from the same male person (with one X and one Y chromosome). Click here for file

Additional file 8 ** Distribution of filtered variants across the genome.** All variants passing a SomaticScore cut-off of 0.5 in the CT (outer circle) and GT analysis (inner circle) are plotted, respectively. SNPs are displayed in orange, insertions in blue, substitutions in green and deletion in yellow. Click here for file

Additional file 9 ** Filtered SNPs unique to the cell line targeting gene loci containing a coding sequence.** Filtered SNPs unique to the cell line targeting gene loci containing a coding sequence. Click here for file

Additional file 10 ** Protocol for the creation of lymphoblastoid cell line.** The detailed protocol that was used to create the lymphoblastoid cell line that was studied. (DOCX 136 kb) Click here for file

Additional file 11 ** Quality of the DNA sent for sequencing.** 400 ng of DNA were loaded per lane on a 1% agarose gel. Marker: 1 kB DNA ladder (New England Biolabs, Ipswich, MA). No DNA degradation was detectable. Samples were run on the same gel, but on opposite sides. Click here for file
