# Supplementary figures and images for: In depth comparison of an individual’s DNA and its lymphoblastoid cell line using whole genome sequencing
Source: BMC Genomics. 2012 Sep 14;13:477. doi: 10.1186/1471-2164-13-477 (PMC3473256; doi:10.1186/1471-2164-13-477)

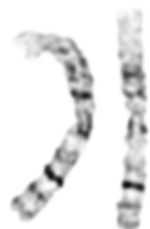

1

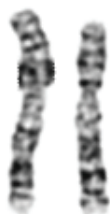

2

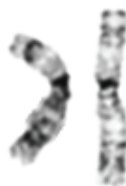

3

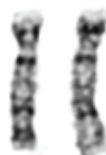

4

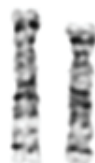

5

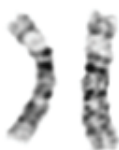

6

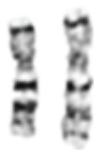

7

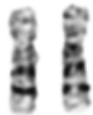

8

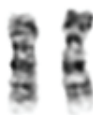

9

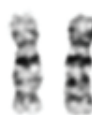

10

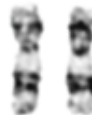

11

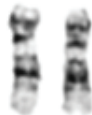

12

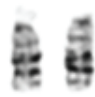

13

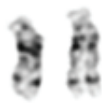

14

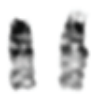

15

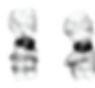

16

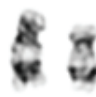

17

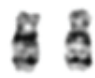

18

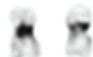

19

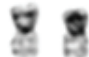

20

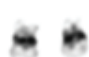

21

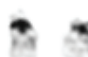

22

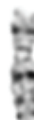

X

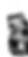

Y

Supplement: Additional file 1 — High resolution karyotype of cell line. The cell line exhibits a normal karyotype. [file 1471-2164-13-477-S1.pdf]

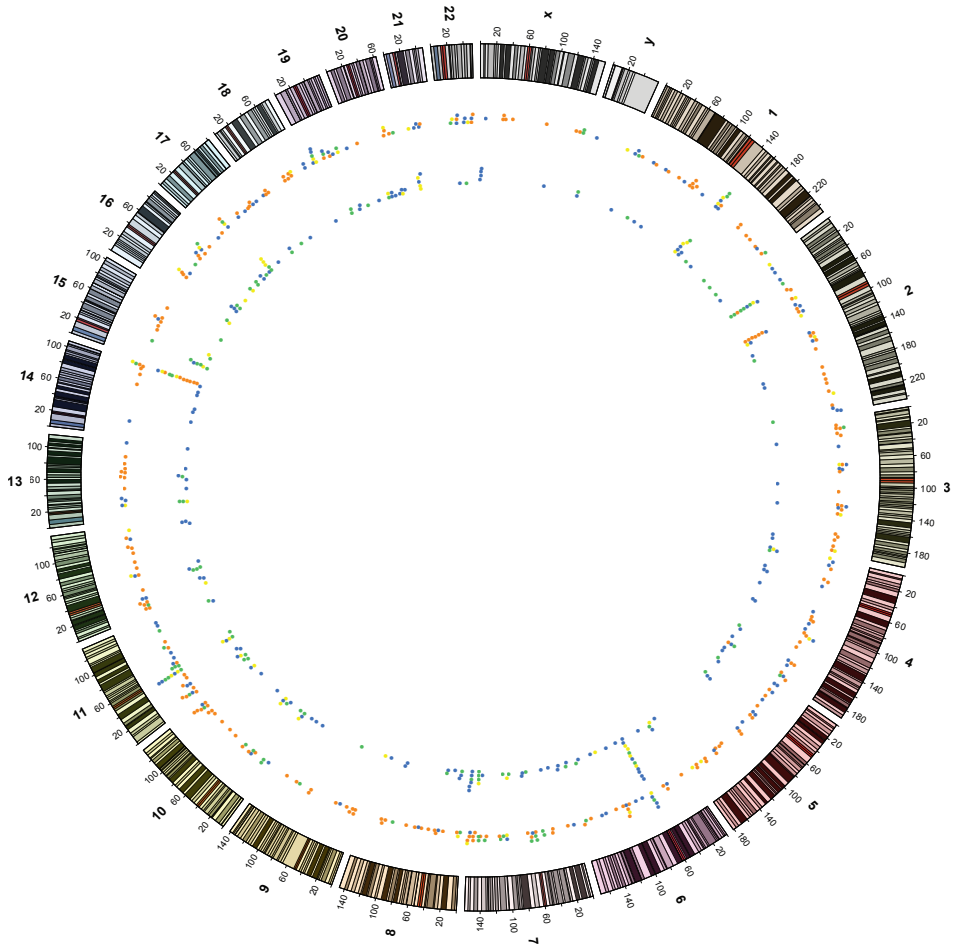

Supplement: Additional file 8 — Distribution of filtered variants across the genome. All variants passing a SomaticScore cut-off of 0.5 in the CT (outer circle) and GT analysis (inner circle) are plotted, respectively. SNPs are displayed in orange, insertions in blue, substitutions in green and deletion in yellow. [file 1471-2164-13-477-S8.pdf]

genomic  
DNA

cell line  
DNA

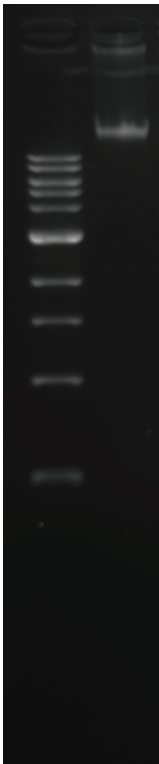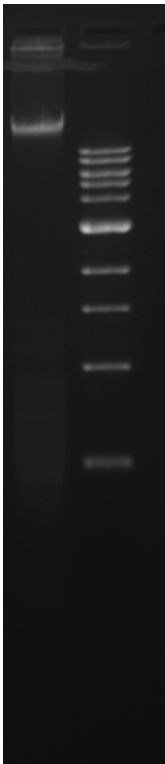

Supplement: Additional file 11 — Quality of the DNA sent for sequencing. 400 ng of DNA were loaded per lane on a 1% agarose gel. Marker: 1 kB DNA ladder (New England Biolabs, Ipswich, MA). No DNA degradation was detectable. Samples were run on the same gel, but on opposite sides. [file 1471-2164-13-477-S11.pdf]
