# Supplementary material for: In depth comparison of an individual’s DNA and its lymphoblastoid cell line using whole genome sequencing
Source: BMC Genomics. 2012 Sep 14;13:477. doi: 10.1186/1471-2164-13-477 (PMC3473256; doi:10.1186/1471-2164-13-477)
Supplement: Additional file 3 — Number of chromosomal rearrangements in both genomes. Information on all high confidence junctions identified per genome and the number of unique junctions, specifying the number of junctions with a frequency of less than or equal to 75% (i.e. seen in less than or exactly 75% of all genomes sequenced by CGI at the time) and the percentage of inter-chromosomal rearrangements. [file 1471-2164-13-477-S3.pdf]

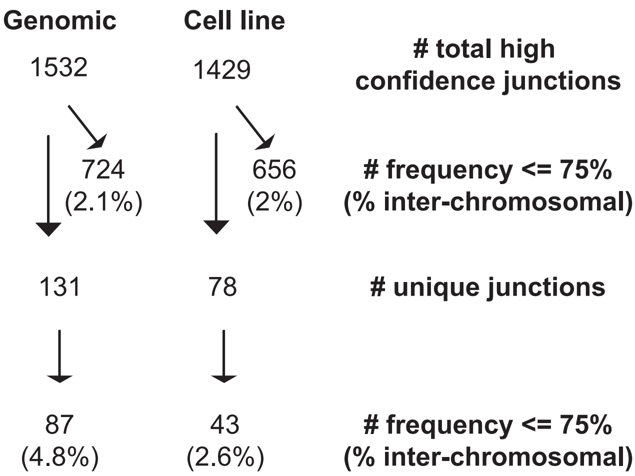

Genomic
Cell line
1532
1429
total high confidence junctions
724 (2.1%)
656 (2%)
frequency <= 75% (% inter-chromosomal)
131
78
unique junctions
87 (4.8%)
43 (2.6%)
frequency <= 75% (% inter-chromosomal)
