# Supplementary material for: In depth comparison of an individual’s DNA and its lymphoblastoid cell line using whole genome sequencing
Source: BMC Genomics. 2012 Sep 14;13:477. doi: 10.1186/1471-2164-13-477 (PMC3473256; doi:10.1186/1471-2164-13-477)
Supplement: Additional file 7 — Analysis strategy and overall differences found between the two sequenced genomes.A. Workflow of the genome analysis. Besides various data files and software tools provided by Complete Genomics (blue), Bioconductor packages and R (green) were used for analysis. B. Gross comparison statistics as output from cgatools calldiff SuperlocusStats. “Identical” sequences are sequences that are fully called and identical in both genomes. “Consistent” sequences are not fully called, but what is called is identical in both genomes. “Only C” and “Only G” denote variants only found in cell line and genomic DNA, respectively. At “mismatch” positions, the two genomes are different from each other and different from the reference. “Phase-mismatch” means that even though the two genomes have the same alleles, the phase of the alleles differ. The two genomes don’t have any “ploidy mismatches” because genomes are from the same male person (with one X and one Y chromosome). [file 1471-2164-13-477-S7.pdf]

A

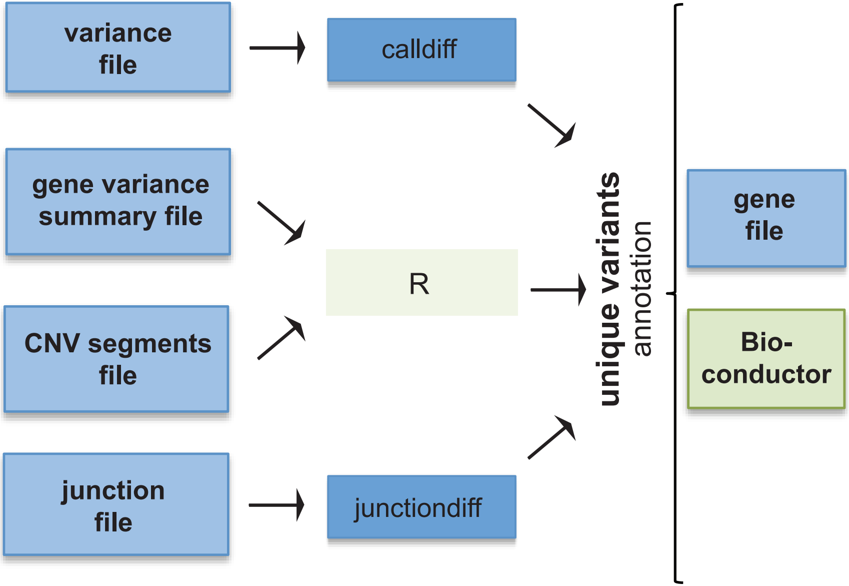

B

Classification	Count	Pct[%]
identical	3218282	84.40
consistent	564205	14.80
only C	11435	0.30
only G	15364	0.40
mismatch	3536	0.09
phase-mismatch	336	0.01
ploidy-mismatch	0	0.00
