# Supplementary material for: In depth comparison of an individual’s DNA and its lymphoblastoid cell line using whole genome sequencing
Source: BMC Genomics. 2012 Sep 14;13:477. doi: 10.1186/1471-2164-13-477 (PMC3473256; doi:10.1186/1471-2164-13-477)
Supplement: Additional file 10 — Protocol for the creation of lymphoblastoid cell line. The detailed protocol that was used to create the lymphoblastoid cell line that was studied. (DOCX 136 kb) [file 1471-2164-13-477-S10.docx]

**Protocol for the generation of lymphoblastoid cell lines**

**1) Preparing Eptein Barr Virus (EBV) supernatant**:

1. Thaw a stock of marmoset B95-8 cells. Wash with 9ml fresh feeding media and spin for 10 min. at 1400RPM at room temperature (RT). Discard supernatant.
2. Do a 2^nd^ wash with 10ml fresh feeding media and spin for 10 min. at 1400RPM at RT. Discard supernatant.
3. Re-suspend B95-8 cells with 5-10ml of fresh feeding media and place in a 25cm tissue culture flask.
4. Continue growing and feeding cells (no more than 20-25ml in a 25cm flask) and expanding them into 75cm tissue culture flasks.
5. Split flasks until you have a desired amount.
6. Once you have a desired amount of cells, be sure to freeze down a few aliquots of cells (preferably in aliquots of 10 million per vial) before continuing with the protocol.
7. When ready to prepare the EBV supernatant, make sure that the 75cm flasks have about 50-75ml of media. Once you have reached this volume, stop changing the feeding media until media turns yellow (should be about 7 days).
8. Then wait another 7 days (without feeding.) At this point, flasks should be concentrated/saturated with cells and media should look lemon yellow.
9. Collect everything (cells and media) from all flasks and fill 50ml falcon tubes—be sure not to top off the tubes. Stop at 45ml line.
10. You are now ready for cell lysis (Freeze-Thaw method)
    1. Place filled tubes into dry ice. Add 95% ethanol (or any other alcohol). This decreases the temperature. Wait until supernatant is frozen.
    2. Thaw the tubes in a water bath set at 37*C.
    3. Repeat 2 more times (a total of 3 freeze-thaws).
    4. Spin tubes at 1400RPM for 15 min. at RT.
    5. Collect supernatant and filter through a 0.45um PES filter.
    6. Aliquot supernatant into tubes (in aliquots of 3ml, 6ml, 9ml, 12ml, etc.—as many of each as you like) and freeze at -80*C.
    7. Do not refreeze supernatant after you thaw to use in transformation. If there is supernatant leftover, discard it.

**2) Establishing a cell line**

1. Isolate lymphocytes using Ficoll gradient.
2. Prepare fresh feeding media.
3. For each 25cm tissue culture flask, add 3.5ml of fresh feeding media. Add 1.5ml of EBV supernatant to lymphocytes, re-suspend, and place cells into flask.
4. Place flasks in incubator in an upright position. Loosen caps to allow air exchange. The incubator should be at 37*C, with CO2 rate of 5%.
5. Do not change media for 7 days. However, at least once during the 7 days, you may want to check the media for contamination. If the flasks look clear, then do not touch for 7 days.
6. On the 7^th^ day, aspirate about 2ml out of the 25cm flask. Be careful not to disturb cells, which have settled to the bottom. Add 2ml of fresh media. If the media looks a bit pinkish, then a boost may be needed. Instead of adding 2 ml of fresh media, add 1ml of EBV supernatant and 1ml of fresh media.
7. Repeat feeding. As cells begin to transform (under the microscope, one can see cell morphology change and cells begin to aggregate), the color of the media begins to turn yellow.
8. Once transformation begins, the cells begin to expand. Increase feeding and add additional media. This should take about 3-4weeks. Once transformed, begin expanding cell lines slowly.
9. When volume of flask reaches 20-25ml, expand cells into a 75cm flask. Do not add too much media—this will shock the cells. Instead, add 5-10ml of media and check the next day. If media looks yellow, then change the media and increase the amount added.
10. Feed cells every other day (about 3 times per week.)
11. When a desired amount of cells is reached, cells can be collected for freezing or harvested for DNA preparation.
